# Role of acid-sensing ion channels in hypoxia- and hypercapnia-induced ventilatory responses

**DOI:** 10.1371/journal.pone.0192724

**Published:** 2018-02-23

**Authors:** Neil D. Detweiler, Kenneth G. Vigil, Thomas C. Resta, Benjimen R. Walker, Nikki L. Jernigan

**Affiliations:** Vascular Physiology Group, Department of Cell Biology and Physiology, University of New Mexico Health Sciences Center, Albuquerque, NM, United States of America; National Yang-Ming University, TAIWAN

## Abstract

Previous reports indicate roles for acid-sensing ion channels (ASICs) in both peripheral and central chemoreception, but the contributions of ASICs to ventilatory drive in conscious, unrestrained animals remain largely unknown. We tested the hypotheses that ASICs contribute to hypoxic- and hypercapnic-ventilatory responses. Blood samples taken from conscious, unrestrained mice chronically instrumented with femoral artery catheters were used to assess arterial O_2_, CO_2_, and pH levels during exposure to inspired gas mixtures designed to cause isocapnic hypoxemia or hypercapnia. Whole-body plethysmography was used to monitor ventilatory parameters in conscious, unrestrained ASIC1, ASIC2, or ASIC3 knockout (^-/-^) and wild-type (WT) mice at baseline, during isocapnic hypoxemia and during hypercapnia. Hypercapnia increased respiratory frequency, tidal volume, and minute ventilation in all groups of mice, but there were no differences between ASIC1^-/-^, ASIC2^-/-^, or ASIC3^-/-^ and WT. Isocapnic hypoxemia also increased respiratory frequency, tidal volume, and minute ventilation in all groups of mice. Minute ventilation in ASIC2^-/-^ mice during isocapnic hypoxemia was significantly lower compared to WT, but there were no differences in the responses to isocapnic hypoxemia between ASIC1^-/-^ or ASIC3^-/-^ compared to WT. Surprisingly, these findings show that loss of individual ASIC subunits does not substantially alter hypercapnic or hypoxic ventilatory responses.

## Introduction

Arterial O_2_, CO_2_, and pH (P_a_O_2_, P_a_CO_2_, and pH_a_) homeostasis is maintained by reflex control of ventilation. Alterations in P_a_O_2,_ P_a_CO_2_, and pH_a_ are detected by peripheral chemoreceptors located in type I glomus cells within the carotid and aortic bodies. P_a_CO_2_ homeostasis is additionally regulated by central chemoreceptors located on the ventral surface of the medulla as well as other brain regions [1]. Activation of carotid chemoreceptors in response to hypoxemia, hypercapnia, or acidosis leads to inhibition of K^+^ channels, depolarization of the chemoreceptor cells, activation of L-type Ca^2+^ channels, and release of excitatory neurotransmitters that subsequently stimulate ventilation and sympathetic activation [2–8]. Although this model of carotid body chemoreception is generally accepted, there are several O_2_- and CO_2_/pH-sensitive ion channels and other proteins and their individual and integrated roles in chemoreception remain incompletely understood. The precise location of *central* chemoreception also remains uncertain, and it appears that several brain regions are involved [1]. A major mechanism by which central chemoreceptors detect CO_2_ levels is thought to be the detection of secondary changes in cerebrospinal fluid pH [1]. Ion channels are generally the favored candidates for central chemoreception [9], but the identity of the specific channels involved remains unclear, with several candidates having been proposed [10–13]. One family of ion channels implicated in both peripheral and central chemoreception is that of the proton-gated, acid-sensing ion channels (ASICs).

ASICs are members of the degenerin\epithelial Na^+^ channel (DEG/ENaC) superfamily that form trimeric cation channels. ASIC genes (ASIC1-4) and their splice variants (ASIC1a, -1b, -2a, -2b, -3, and -4) form homo- or hetero-multimeric channels with different pH sensitivities. ASIC1 and 3 homomers are the most sensitive to acidic conditions with a half-maximal activation (pH_50_) of around pH 6.5 [14,15]; whereas ASIC2 is least acid-sensitive (pH_50_ ~ 4.9) [15]. ASIC1 and 3, and to a lesser extent ASIC2, are expressed in glomus cells and the transient acid-evoked depolarization of isolated glomus cells is consistent with the biophysical and pharmacological properties of ASICs [16]. ASICs are also widely expressed in the medulla where reductions in pH trigger ASIC-like currents and stimulation of phrenic nerve activity in anesthetized animals [17–19]. Based on these reports that suggest that ASICs play an important role in chemoreception, we hypothesized that ASICs contribute to hypercapnic/acidosis-induced ventilatory drive in conscious, unrestrained mice.

Recent studies from our laboratory also demonstrate that ASIC1, expressed in pulmonary arterial smooth muscle cells (PASMC), contributes to hypoxic pulmonary vasoconstriction (HPV) [20]. Although the precise mechanism remains unclear, HPV is generally thought to be an intrinsic response of O_2_-sensing PASMC, supporting ventilation-perfusion matching for optimal gas exchange in the lung by diverting blood flow away from hypoxic regions of the lung_._ Our data showing reduced HPV in ASIC1 null mice [20] suggest ASICs are sensitive to changes in O_2._ Therefore, we further hypothesized that ASICs additionally contribute to the *hypoxic* ventilatory response. To test our hypotheses, we examined ventilatory responses to isocapnic hypoxemia and hypercapnia in conscious, unrestrained, ASIC1, 2, or 3 global knockout (ASIC1^-/-^, 2^-/-^, or 3^-/-^) and wild-type (WT) mice.

## Materials and methods

### Animals

All protocols used in this study were reviewed and approved by the Institutional Animal Care and Use Committee of the University of New Mexico School of Medicine (Protocol number: 16-200543-HSC) and abide by the National Institutes of Health guidelines for animal use. All surgeries (described below) were performed under isoflurane anesthesia and buprenorphine was used for post-operative analgesia. ASIC1 (B6.129-Asic1^tm1Wsh^/J), ASIC2 (B6.129-Asic2^tm1Wsh^/J), and ASIC3 (B6.129-Asic3^tm1Wsh^/J) knockout (^-/-^) mice (all from Jackson Laboratory, Bar Harbor, ME) were bred on a C57BL/6 background and compared to age-matched C57BL/6 wildtype (WT) controls. Disruption of the relevant ASIC was confirmed by PCR and agarose gel electrophoresis using a three-primer (ASIC1^-/-^ and ASIC2^-/-^) or four-primer (ASIC3^-/-^) system to detect both WT and disrupted alleles. The following primers were used for genotyping: ASIC1: 5’-CAT GTC ACC AAG CTC GAC GAG GTG-3’ (WT forward primer), 5’-TGG ATG TGG AAT GTG TGC GA-3’ (knockout forward primer), 5’-CCG CCT TGA GCGGCA GGT TTA AAG G-3’ (reverse primer); ASIC2: 5’-AGT CCT GCA CGG TGG GAG CTT CTA-3’ (reverse primer) 5’-GAA GAG GAA GGG AGC CAT GAT GAG-3’ (WT forward primer), 5’-TGG ATG TGG AAT GTG TGC GA-3’ (knockout forward primer); ASIC3: 5’-GAA CCT GGA AAA CAG AGG CAG GAA GGA T-3’ (knockout reverse primer), 5’-CAG GGA GTA AGA TCT TAT GTA GCC TGG C-3’ (knockout forward primer), 5’-TGG ATG TGG AAT GTG TGC GA-3’ (WT reverse primer), 5’-CCC TGG GCA CCA GAG TTG AAG GTG TAG C-3’ (WT forward primer).Males and females (~15 wk old) were used equally. Each group of knockout mice was paired with a separate, simultaneously run set of WT mice.

### Femoral artery catheterization and blood gas measurement

To confirm that alterations in inspired gases were achieving the desired effect on blood gases, and that blood gases were similar between paired groups of WT and knockouts, mice were chronically instrumented with femoral artery catheters for arterial blood sampling to determine P_a_O_2_, P_a_CO_2_, and pH_a_. The catheters were routed out through the top of the cage through spring tethers to enable blood sampling in conscious, unrestrained mice. Catheters consisted of PE-10 tubing with a stretch-tapered proximal end for insertion into the right femoral artery, and were filled with a solution of 0.9% saline containing 100 units/ml heparin. Catheterization was performed under isoflurane anesthesia (5% isoflurane for induction of anesthesia, ~2% for maintenance) and mice were given buprenorphine (0.05–0.1 mg/kg, s.c.) and enrofloxacin (15 mg/kg, s.c.) post-operatively for analgesia and protection from infection, respectively.

Blood gas measurements were performed 2 days after the implantation surgery. For these experiments, mice were placed in a polycarbonate chamber for exposure to different inspired gas mixtures. A Columbus Instruments PEGAS 4000MF gas mixer (Columbus, OH) was used to combine N_2_, O_2_, and CO_2_ for the appropriate inspired gas mixtures, the compositions of which were confirmed using an OxiGraf O_2_Cap Oxygen [and CO_2_] Analyzer (Sunnyvale, CA) to test samples taken from chamber inflow line. To measure blood gases, ~100 μL of blood was allowed to flow directly from the femoral artery catheter into Abbott iStat handheld blood gas analyzer cartridges (EG6+ or G3+, Abbott Park, IL) while the mouse was conscious and unrestrained. Each measurement was taken after 5 minutes of exposure to the respective inspired gas mixture, with at least 20 minutes between subsequent exposures. The catheter was flushed with a volume of saline (containing 100 units/mL heparin) equivalent to 1.5 times the dead space of the catheter between each measurement. For each individual mouse, all blood gas measurements were performed on a single day.

### Whole-body plethysmography

Whole-body plethysmography was used to assess respiratory frequency, tidal volume, and minute ventilation in conscious, unrestrained mice. First described by Drorbaugh and Fenn [21], this method utilizes a nearly sealed chamber in which the pressure transiently increases as inspired air expands due to the humidity and warmth of the lung compared to the surrounding environment. For this study we modified a method previously used for rats [22]. The plethysmography chamber consisted of a ~50 cubic centimeter transparent polycarbonate cylinder fitted with a Validyne DP45-16 differential pressure transducer and inlets for the introduction of new inspired gas mixtures. To take a measurement, the chamber was sealed with the exception of a small controlled leak through a 50 μl glass syringe (Gastight #1705) with plunger removed, attached to one of the Luer-lock ports on the side of the chamber, which served as a physical high-pass filter to eliminate gradual changes in chamber pressure that go beyond the narrow range of the highly sensitive differential pressure transducer. Temperature inside the chamber was monitored using an electronic probe (BAT-10, Physitemp, Clifton, NJ) and ranged from 21 to 25° C. Calibration air injections of 40 μl were used to enable calculation of tidal volume using the equation introduced by Drorbaugh and Fenn [21]:

$$V_{T}=\frac{P_{T}}{P_{K}}\times V_{K}\times \frac{T_{R}(P_{B}-P_{C})}{T_{R}(P_{B}-P_{C})-T_{C}(P_{B}-P_{R})}$$

Where:

V_T_ = tidal volume

V_K_ = the volume of air injected into the animal chamber for calibration

P_T_ = the pressure deflection associated with each tidal volume

P_K_ = the pressure deflection associated with injection of the calibrating volume, V_K_

T_R_ = body temperature, assumed to be 37°C for all animals

T_C_ = the air temperature in the chamber, which varied from 20–23°C

P_B_ = barometric pressure, 630 mmHg in Albuquerque, NM

P_R_ = vapor pressure of water at body temperature

P_C_ = vapor pressure of water in the chamber, derived from T_C_ assuming 100% humidity which was confirmed in pilot experiments (gas mixtures were humidified by bubbling through three consecutive flasks of water prior to entry into the plethysmography chamber)

Tidal volume and minute ventilation data were normalized to body mass. The plethysmography chamber was never closed from gas flow for measurement for more than 2 consecutive minutes, and the chamber was continuously flushed with fresh gas mixture for at least 2 minutes between measurements at a flow rate of 2 L/min. Mice were allowed up to 1 hour to achieve a resting state suitable for baseline measurements (lack of movement, grooming, or sniffing as observed through the transparent walls of the chamber). Mice that remained active after 1 hour were removed from the chamber and re-tested on another day. After a baseline measurement was taken, the chamber was flushed with 21% O_2_, 6% CO_2_, balance N_2_ to achieve hypercapnia, or 7% O_2_, 3.2% CO_2_, balance N_2_ to achieve isocapnic hypoxemia for 5 minutes before taking the subsequent measurement.

### Statistics

All data are expressed as means ± SE. n values correspond to the numbers of animals per group. Statistical tests are specified in the figure legends or in the results section, and were made using GraphPad Prism 7.02 software (La Jolla, CA). *P* values of <0.05 were accepted as significant for all comparisons.

## Results

### Examining the roles of ASIC1, 2, and 3 in the hypercapnic ventilatory response

**Fig 1:**
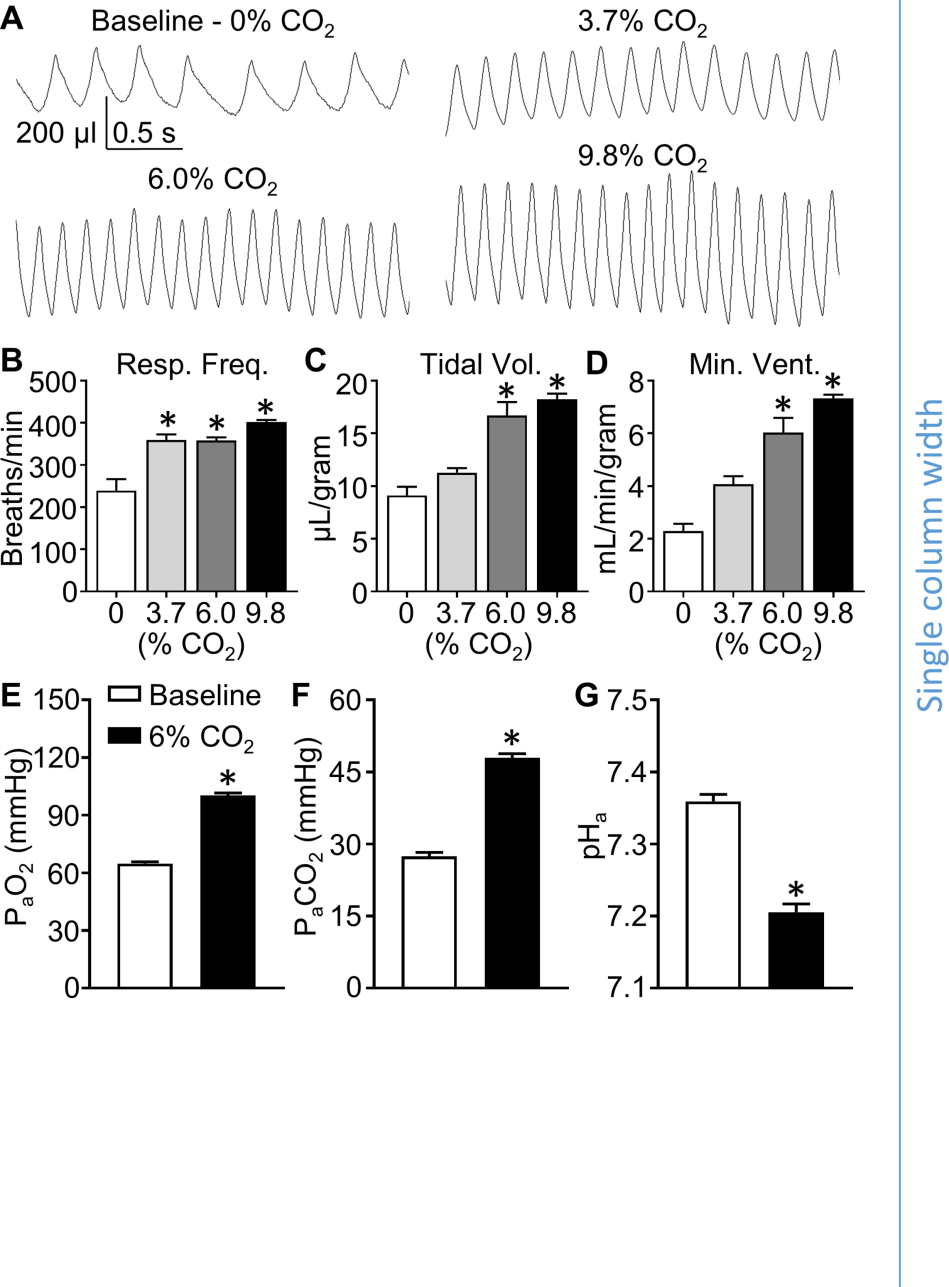
Inhalation of 6% CO_2_ produces reliable hypercapnia and elevation of ventilation. WT mice were exposed to a range of inspired CO_2_ levels to determine optimal conditions for testing hypercapnic ventilatory responses. **(A)** Representative traces of whole-body plethysmography illustrate frequency and depth of breathing in conscious, unrestrained mice exposed to normal air at baseline (21% O_2_, 0% CO_2_, balance N_2_), and to increasing levels of hypercapnia (3.7, 6.0, and 9.8% CO_2_). Summary data shows **(B)** respiratory frequency (breaths/min), **(C)** tidal volume (μL/breath/g), and **(D)** minute ventilation (mL/min/g) at each inspired CO_2_ level. Arterial blood samples taken from WT mice at baseline (normal air) or during exposure to 6% CO_2_ were analyzed for **(E)** P_a_O_2,_ **(F)** P_a_CO_2_, and **(G)** pH_a_. Values are means ± SE; n = 4–8 animals/group. *P < 0.05 vs. baseline (1-way ANOVA; Dunnett’s post-hoc test (B) or two-tailed, paired Student’s t-test (C-E)).

To investigate the roles of ASIC1, 2, and 3 in the hypercapnic ventilatory response, we first assessed changes in ventilation during exposure to increasing levels of inspired CO_2_ (3.7, 6.0, and 9.8%) using whole-body plethysmography in conscious, unrestrained WT mice. The traces shown in Fig 1A represent the pressure difference between the inside and the outside (room pressure) of the chamber. Upward pressure defections indicate increasing pressure in the animal chamber (inspiration). The amplitude of the pressure deflections corresponds to tidal volume. Respiratory frequency (Fig 1B, breaths/min) was determined by counting the number of pressure defections per minute. Exact tidal volume normalized to body mass (Fig 1C, μL/breath/g) was calculated using chamber temperature and calibration air injection (see Methods). Minute ventilation normalized to body mass (Fig 1D, mL/minute/gram) was determined by multiplying tidal volume by respiratory frequency.

First, we measured minute ventilation in mice exposed to 5 minutes of 3 levels of inspired CO_2_ (3.7, 6.0, and 9.8%) to determine an appropriate level for testing hypercapnic ventilatory responses (Fig 1A–1D). In these pilot experiments, exposure to 3.7%, 6.0%, and 9.8% inspired CO_2_ significantly elevated respiratory frequency (Fig 1B), but only exposure to 6.0% and 9.8%, and not 3.7% inspired CO_2_ caused statistically significant elevations in tidal volume and minute ventilation (Fig 1C and 1D, respectively). We chose to use 6% CO_2_ as the hypercapnic stimulus for the remaining experiments in this study because it stimulated a robust response that was not statistically different from the response caused by 9.8% CO_2_. Using arterial blood samples obtained via chronically implanted femoral artery catheters in conscious, unrestrained WT mice, we assessed P_a_O_2_, P_a_CO_2_, and pH_a_ at baseline (room air) and during exposure to 6% inspired CO_2_. Although inspired O_2_ was unchanged (21%) during exposure to 6% inspired CO_2_, P_a_O_2_ was increased (Fig 1E) likely due to the resultant increase in alveolar ventilation. As expected, mice exhibited hypercapnia and acidosis indicated by increased P_a_CO_2_ and decreased pH_a_ during exposure to 6% inspired CO_2_ (Fig 1F and 1G).

**Fig 2:**
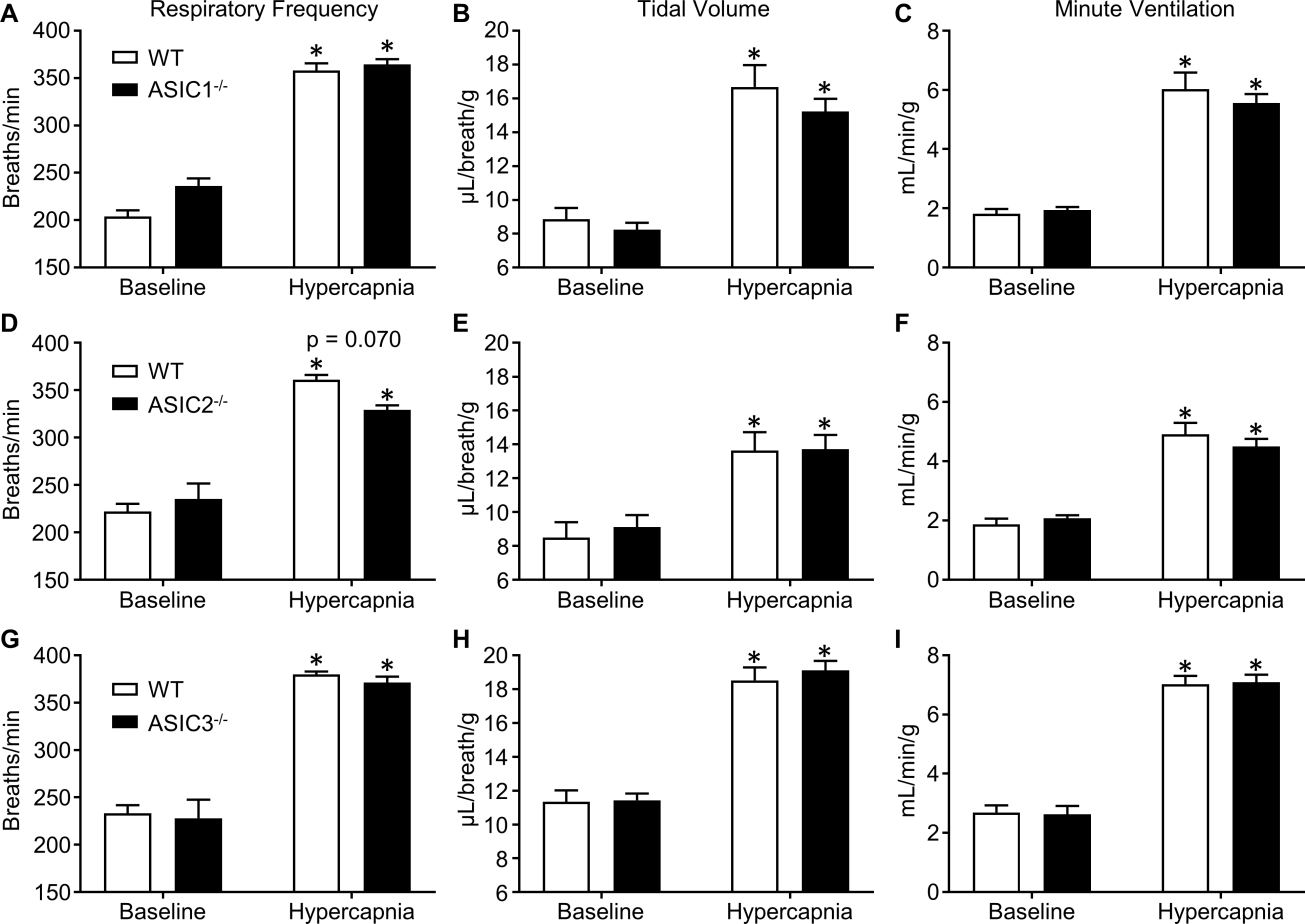
The hypercapnic ventilatory response does not require ASIC1, 2, or 3. Whole-body plethysmography was used to determine respiratory frequency (breaths/min; A, D, G), tidal volume (μL/breath/g; B, E, H), and minute ventilation (ml/min/g body wt; C, F, I) at baseline and during exposure to hypercapnia (6% CO_2_) in WT, ASIC1^-/-^ (A-C), ASIC2^-/-^ (D-F), and ASIC3^-/-^ mice (G-I). Values are means ± SE; n = 8 (A-C), 7–8 (D-F), or 11–12 (G-I) animals/group. *P < 0.05 vs. baseline (2-way, repeated measures ANOVA; Sidak’s post-hoc test). Displayed P value corresponds to comparison between WT and KO (panel D).

Next, we measured P_a_O_2_, P_a_CO_2_, and pH_a_ in ASIC1^-/-^, ASIC2^-/-^, ASIC3^-/-^, and paired groups of WT mice. The purpose of this experiment was to determine if the stimuli for ventilation (P_a_O_2_, P_a_CO_2_, and pH_a_) were the same between WT and knockout mice. Our results show no significant differences in P_a_O_2_, P_a_CO_2_, and pH_a_ between ASIC1^-/-^, ASIC2^-/-^, or ASIC3^-/-^ versus corresponding WT mice at baseline or during exposure to 6% inspired CO_2_ (Table 1). To investigate the putative roles of ASIC1, ASIC2, and ASIC3 in the hypercapnic/acidotic ventilatory response, we exposed separate groups of ASIC1^-/-^, ASIC2^-/-^, and ASIC3^-/-^ mice to 6% inspired CO_2_ and measured respiratory frequency, tidal volume, and minute ventilation using whole-body plethysmography as described above, and compared the responses to those of WT mice run in parallel for each experiment. Surprisingly, there were no significant differences between ASIC1^-/-^, ASIC2^-/-^, or ASIC3^-/-^ mice compared to WT mice in respiratory frequency, tidal volume, or minute ventilation (Fig 2). There was a tendency for ASIC2^-/-^ mice to exhibit lower respiratory frequency during hypercapnia (Fig 2D), but this trend was not statistically significant (p = 0.070).

**Table 1:** Blood gas levels were not different between paired WT and knockout groups at baseline, during hypercapnia, or during isocapnic hypoxemia.

**Baseline (21% O**_**2**_**, 0% CO**_**2**_**, balance N**_**2**_**)**						
	WT	ASIC1^-/-^	WT	ASIC2^-/-^	WT	ASIC3^-/-^
P_a_O_2_	63.2 ± 0.9	66.4 ± 0.8	58.6 ± 1.0	62.1 ± 2.2	64.9 ± 0.9	70.9 ± 2.7
P_a_CO_2_	28.0 ± 1.1	26.2 ± 0.5	28.4 ± 0.3	29.3 ± 1.0	27.4 ± 0.8	26.7 ± 1.2
pH_a_	7.37 ± 0.01	7.39 ± 0.01	7.38 ± 0.01	7.39 ± 0.01	7.36 ± 0.01	7.34 ± 0.02
**Hypercapnia (21% O**_**2**_**, 6.0% CO**_**2**_**, balance N**_**2**_**)**						
	WT	ASIC1^-/-^	WT	ASIC2^-/-^	WT	ASIC3^-/-^
P_a_O_2_	100.2 ± 2.1*	99.6 ± 2.1*	90.3 ± 1.2*	95.9 ± 2.9*	100.1 ± 1.4*	103.6 ± 3.5*
P_a_CO_2_	45.9 ± 1.5*	45.8 ± 0.8*	44.8 ± 0.7*	45.5 ± 0.9*	47.9 ± 0.9*	45.8 ± 0.9*
pH_a_	7.23 ± 0.02*	7.22 ± 0.01*	7.23 ± 0.01*	7.24 ± 0.02*	7.21 ± 0.01*	7.20 ± 0.02*
**Isocapnic hypoxemia (7.0% O**_**2**_**, 3.2% CO**_**2**_**, balance N**_**2**_**)**						
	WT	ASIC1^-/-^	WT	ASIC2^-/-^	WT	ASIC3^-/-^
P_a_O_2_	40.3 ± 1.5*	40.4 ± 0.6*	35.6 ± 1.2*	38.5 ± 1.4*	39.3 ± 0.8*	42.7 ± 1.5*
P_a_CO_2_	25.5 ± 0.8*	25.1 ± 0.5	25.6 ± 0.4*	26.1 ± 0.7*	28.0 ± 0.6	27.3 ± 0.6
pH_a_	7.39 ± 0.02	7.39 ± 0.00	7.40 ± 0.01	7.40 ± 0.02	7.35 ± 0.02	7.32 ± 0.2

Arterial blood gas levels were assessed in samples taken from conscious, unrestrained mice chronically instrumented with femoral artery catheters at baseline (room air) or during exposure to 6% inspired CO_2_ or 7% O_2_, 3.2% CO_2_ to induce hypercapnia or isocapnic hypoxemia, respectively. No differences in P_a_O_2_, P_a_CO_2_, or pH_a_ between paired WT and knockout groups were detected. Values are means ± SE; n = 5–8 animals/group.

*P < 0.05 vs. baseline (2-way, repeated measures ANOVA; Sidak’s post-hoc test).

### Assessing the roles of ASIC1, 2, and 3 in the hypoxic ventilatory response

**Fig 3:**
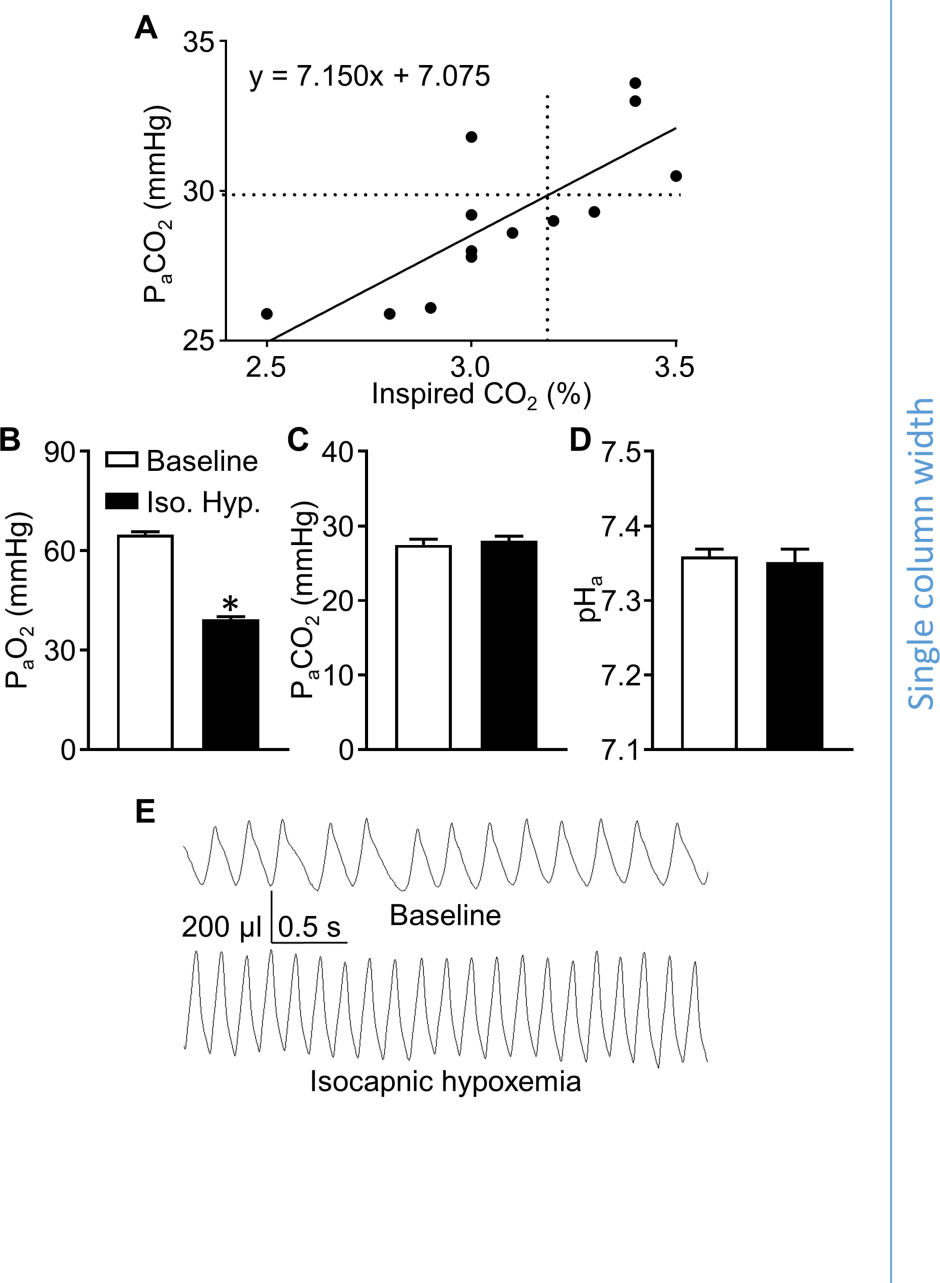
An inspired CO_2_ of 3.2% causes isocapnic hypoxemia in mice concurrently exposed to 7.0% O_2_. A scatter plot **(A)** showing arterial CO_2_ tensions (P_a_CO_2_; y-axis) in mice exposed to 7.0% O_2_ and a range of inspired CO_2_ levels (% CO_2_; x-axis) was fit using linear regression (solid line, equation displayed on the graph). The dotted lines on the graph indicate the average baseline P_a_CO_2_ (horizontal) measured during exposure to room air, and the estimated inspired CO_2_ required to maintain this P_a_CO_2_ level (vertical) during exposure to 7% O_2_ based on the linear regression. Arterial blood samples from WT mice exposed to 7.0% O_2_, 3.2% CO_2_, balance N_2_, were analyzed to assess P_a_O_2_ **(B)**, P_a_CO_2_ **(C)**, and pH_a_ **(D)**. **(E)** Representative whole-body plethysmography traces from a WT mouse show the ventilatory response to 7.0% O_2_, 3.2% CO_2_, balance N_2_. Values are individual measurements (A) or means ± SE (B-D); n = 5–6 animals per group (B-D). *P < 0.05 vs. baseline (paired, two-tailed Student’s t-test).

To specifically assess the roles of ASICs in the ventilatory response to hypoxia, we measured responses to isocapnic hypoxemia, which was achieved via inhalation of a hypoxic gas mixture with a level of inspired CO_2_ adequate to preserve baseline P_a_CO_2_ levels, allowing for the assessment of the hypoxic ventilatory response without confounding changes in P_a_CO_2_/pH_a_-dependent ventilatory drive. To determine the level of inspired CO_2_ required to achieve isocapnic hypoxemia, we measured baseline P_a_CO_2_ in WT animals, and then experimentally determined the level of inspired CO_2_ necessary to maintain this P_a_CO_2_ in mice concurrently exposed to 7% inspired O_2_. Baseline P_a_CO_2_ in WT mice was 29.8 ± 0.9 mmHg. Mice chronically instrumented with femoral artery catheters were exposed to 7% O_2_ at a range of inspired CO_2_ levels (2.5–3.5%) and the resulting P_a_CO_2_ was assessed (Fig 3A). Linear regression analysis of these data indicate that an inspired CO_2_ of 3.2% produces isocapnic hypoxemia. Testing this exposure in a group of WT mice (n = 7) confirmed significant hypoxemia (Fig 3B) with no accompanying change in P_a_CO_2_ or pH_a_ (Fig 3C and 3D). Whole-body plethysmography measurements show that this exposure robustly increased respiratory frequency and tidal volume (Fig 3E). We used this stimulus (7% O_2_, 3.2% CO_2_, balance N_2_) to compare hypoxic ventilatory responses in the following experiments.

**Fig 4:**
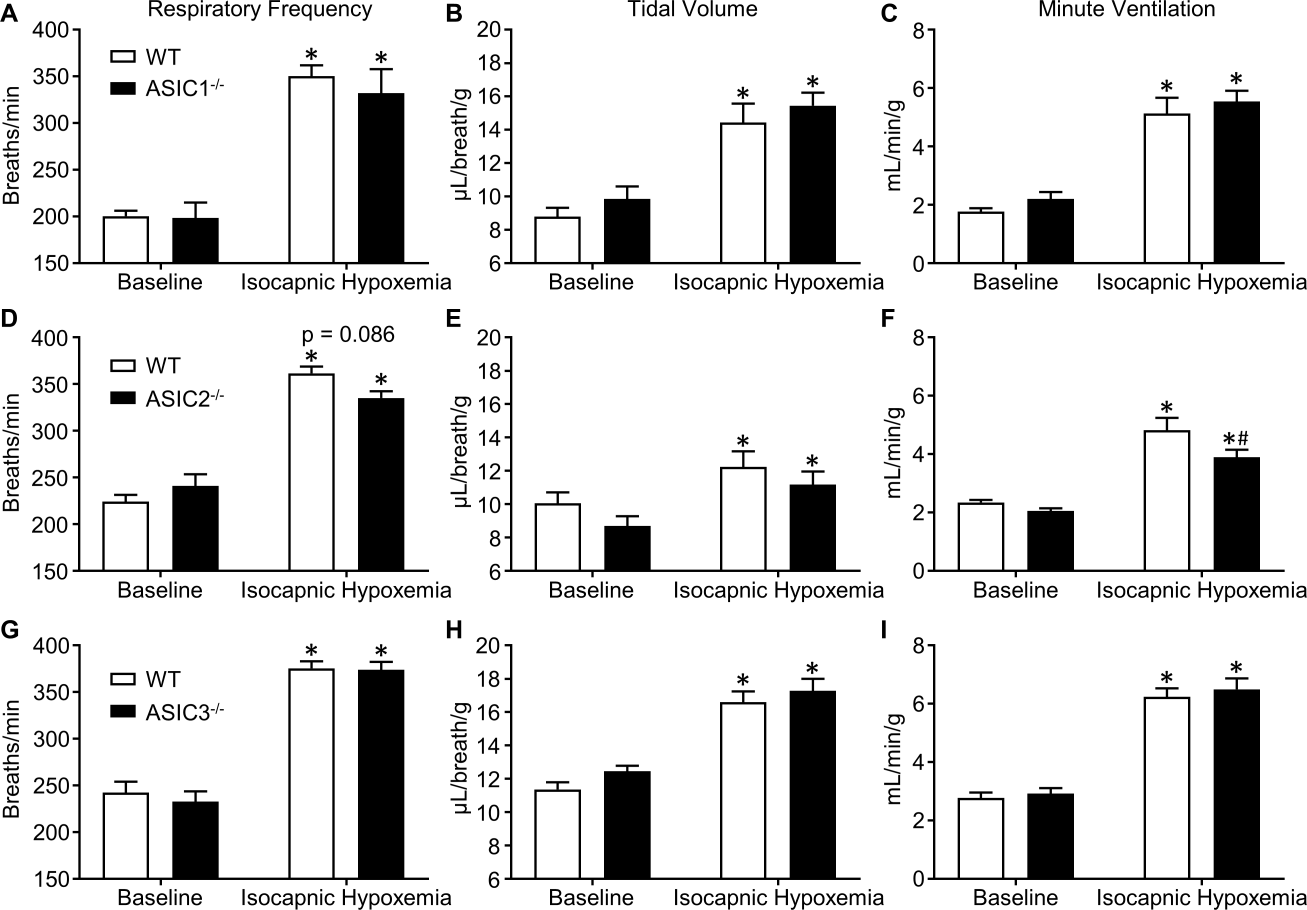
ASIC2, but not ASIC1 or ASIC3, contributes to the hypoxic ventilatory response. Ventilatory responses to isocapnic hypoxemia (7.0% O_2_, 3.2% CO_2_, bal N_2_) were assessed using whole-body plethysmography and compared between **(A-C)** ASIC1^-/-^, **(D-F)** ASIC2^-/-^, and **(G-I)** ASIC3^-/-^ and WT mice. Values are means ± SE; n = 8 (A-F) or 11–12 (G-I) animals/group. *P < 0.05 vs. baseline; ^#^*P* < 0.05 vs. WT (2-way, repeated measures ANOVA; Sidak’s post-hoc test). Displayed P value corresponds to the comparison between WT and KO (panel D).

Using ASIC1^-/-^, ASIC2^-/-^, and ASIC3^-/-^, and WT mice chronically instrumented with femoral artery catheters, we measured P_a_O_2_, P_a_CO_2_, and pH_a_ to confirm that there were no differences in blood gases between respective knockout and WT mice at baseline or during exposure to 7% O_2_, 3.2% CO_2_, balance N_2_ (Table 1), demonstrating that a similar hypoxic stimulus was achieved between groups. Although a slight but significant decrease in P_a_CO_2_ occurred in response to this inspired gas mixture compared to baseline in some groups (marked with asterisks, Table 1), in all cases there were no significant differences between paired WT and knockout groups. To test the roles of ASIC1, 2, and 3 in the hypoxic ventilatory response, separate groups of conscious, unrestrained ASIC1^-/-^, ASIC2^-/-^, ASIC3^-/-^, and WT mice were exposed to 7% O_2_, 3.2% CO_2_, balance N_2_ to induce isocapnic hypoxemia and their respiratory frequencies, tidal volumes, and minute ventilations were assessed using whole-body plethysmography (Fig 4). There were no differences in ventilatory parameters between ASIC1^-/-^ or ASIC3^-/-^ and WT mice (Fig 4A–4C and 4G–4I). ASIC2^-/-^ mice tended to have lower respiratory frequency and tidal volume than WT mice during exposure to isocapnic hypoxemia (Fig 4D and 4E). While these trends in respiratory frequency and tidal volume were not statistically significant, minute ventilation was significantly lower in ASIC2^-/-^ mice compared to WT (Fig 4F), suggesting a role for ASIC2 in the hypoxic ventilatory response.

## Discussion

Previous evidence indicates that ASICs are involved in chemoreception and the control of ventilation [16–19,23,24], but most of this evidence comes from *in vitro* studies and little is known about the role of ASICs in hypercapnic and hypoxic ventilatory responses in conscious animals. Here we directly measured hypercapnic and hypoxic ventilatory responses in conscious, unrestrained mice. In contrast to our hypothesis, we found that genetic deletion of ASIC1, 2, or 3 does not alter the hypercapnic ventilatory response in mice; whereas ASIC2 appears to play a minor role in the hypoxic ventilatory response. Together, these data suggest the contribution of ASICs to ventilatory control is modest.

ASICs have been implicated in both peripheral and central CO_2_ chemoreception. Isolated carotid body glomus type I cells exhibit acid (pH 6.0–6.8)-induced cation currents and depolarization which have been attributed to ASICs [16]. Further investigation revealed that pH-sensitivity in isolated glomus cells is increased in both transgenic ASIC3 overexpressing mice and spontaneously hypertensive rats with increased expression of ASIC3, and decreased in ASIC3 null mice [23,24]. These studies suggest that ASIC3 would contribute to hypercapnic ventilatory drive. However, the pH levels used to stimulate a response in these *in vitro* experiments ranged from pH 6.0 to 6.8. In the present study, exposure to 6% inspired CO_2_ caused a 70% increase in P_a_CO_2_, resulting in a pH_a_ of 7.2. This stimulus caused a robust ventilatory response (Figs 1 and 2), yet a pH_a_ of 7.2 may be insufficient to activate ASICs.

Other reports implicate ASICs in *central* chemoreception. The response of central chemoreceptors to changes in pH is known to be very sensitive, in that changes in cerebrospinal fluid pH from 7.30 to 7.25 cause a doubling in alveolar ventilation [25]. In neurons of the nucleus tractus solitarius in brainstem slices, acidification to pH 7.0 causes transient inward currents that are blocked by amiloride [18]. Although amiloride is a non-selective blocker of ASICs, Song et al., found that the selective ASIC1a blocker, psalmotoxin-1 (PcTX1) inhibited the increased phrenic nerve discharge in response to injection of acidified (pH 6.5) artificial cerebrospinal fluid in the lateral hypothalamus [17]. Furthermore, these authors report ASIC-like acid-induced currents in the ventrolateral medulla were absent in ASIC1^-/-^ mice [19]. These data provide strong evidence that ASIC1 contributes to central chemoreception in response to direct changes in pH (pH 6.0–7.0), however whether the observed effect is relevant to the physiological ventilatory response to hypercapnia is less clear. The threshold for activation of ASIC1 and ASIC3 is ~ pH 7.0–7.2 [26,27] and the half-maximal activation is 6.2–6.8 for ASIC1a, ~4.9 for ASIC2a, and 6.5–6.7 for ASIC3 [26,28]. Interestingly, however, Ziemann et al. reported that inhalation of higher CO_2_ concentrations (10 and 20%) in anesthetized mice leads to a drop in pH ranging from 6.7–6.9 in the amygdala and lateral ventricle [29]. Consistent with our findings, the authors demonstrated an intact ventilatory response to CO_2_ inhalation (5 and 10%) in ASIC1^-/-^ mice despite the important chemosensory role ASIC1 plays in the amygdala and fear-related behavior [29]. Acid-evoked ASIC responses in isolated cells tend to be transient, on the order of seconds, while the ventilatory response to hypercapnia is sustained indefinitely. This might explain why the previously reported contribution of ASIC3 to transient acid-induced currents in isolated glomus cells [16,24] does not translate to a contribution to the hypercapnic ventilatory response. However, the studies implicating ASICs in central chemoreception reported sustained changes to phrenic nerve discharge [17–19], suggesting that ASICs can mediate sustained responses to extracellular acidification in some contexts.

Thus, despite strong evidence for ASICs in chemoreception, our current findings do not support a role for ASICs in the hypercapnic ventilatory response in conscious, unrestrained mice. Although differences in ventilatory regulation may exist between species, mice are widely used in ventilatory function studies as they show dependency both on carotid bodies [30] as well as central chemoreception [1] for control of ventilation. Furthermore, mice were used to study the involvement of ASICs in carotid body glomus cells [24] and central chemoreception [19]. Therefore, it is unlikely that our current findings are due to species differences in control of ventilation. Inhalation of 6% CO_2_ was chosen as the stimulus for hypercapnic ventilatory responses based on data presented in Fig 1 which indicates that 6% CO_2_ causes a near-maximal ventilatory response. Although we cannot exclude the potential for ASICs to participate in hypercapnic ventilatory responses to higher levels of CO_2_, this seems unlikely since the response of WT mice to 9.7% CO_2_ was not different compared to the response to 6% CO_2_. Additionally, our results agree with the findings of Ziemann et al. [29] which indicated that ASIC1 was not involved in ventilatory responses to 5% or 10% inspired CO_2_.

Although much of the evidence supporting ASIC involvement in chemoreception focuses on the detection and ventilatory response to CO_2_ or acidosis, evidence suggests ASICs may also be hypoxia-sensitive. For example, we have previously reported that ASIC1 contributes to HPV in isolated-perfused lungs [20]. Additionally, activation of the Ca^2+^-permeable ASIC1a contributes to neuronal cell death during ischemic brain injury [31,32]. Currently however, it is unclear whether the activation of ASICs responsible for neuronal cell death in this context results from the associated acidosis, or a direct effect of hypoxia. Liu et al reported that the hypoxia-induced chemoreceptor response and ASIC expression are both increased following exposure to *chronic* hypobaric hypoxia (380 Torr ambient air pressure for 1–7 days) in the rat petrosal ganglion, which contains chemoafferent neurons that innervate O_2_-sensitive carotid body glomus type I cells [33]. Pharmacological inhibition of ASICs using A-317576 (novel ASIC blocker [34]) or ibuprofen (non-specific ASIC inhibitor [35]) prevented the enhanced hypoxia-evoked chemoreceptor response following exposure of rats to chronic hypoxia, but did not alter the hypoxic chemoreceptor response in normoxia-exposed control animals [33]. In contrast, Lu et al. demonstrated that transgenic overexpression of ASIC3 leads to decreased glomus cell sensitivity to sodium cyanide (used to mimic hypoxia) [24]. Together, these data are consistent with the hypothesis that ASICs modulates hypoxic chemotransmission between type I cells and chemoafferent neurons. In the present study, we have differentiated between hypoxia and acidosis by utilizing *isocapnic* hypoxemia as a ventilatory stimulus that maintains baseline CO_2_ and pH levels. Ventilatory responses to isocapnic hypoxemia were normal in ASIC1^-/-^ and ASIC3^-/-^ mice, but reduced in ASIC2^-/-^ mice (Fig 4F) suggesting a minor role for ASIC2 in this response. ASIC2a was reported to be expressed in carotid body glomus cells [16], and may contribute to the hypoxic ventilatory response by contributing to the depolarization of these cells during hypoxemia.

In summary, our data bring into question the physiological relevance of ASICs in chemoreception and the control of ventilation. We cannot rule out the possibility that compensatory upregulation of parallel chemoreception and ventilatory control pathways masks any effect of ASIC deletion in our global knockout mice. Furthermore, cerebral ischemia or other pathophysiological conditions might unmask a contribution of ASICs to hypercapnic and/or hypoxic ventilatory drive. Because ASICs are potential therapeutic targets for the treatment of pulmonary hypertension [20] and several neuronal diseases [36], a complete understanding of the roles ASICs play in O_2_ and CO_2_ homeostasis is important to the development of safe therapeutics.
